# Analysis of methylation features associated with neoadjuvant efficacy in HER2-positive breast cancer

**DOI:** 10.3389/fonc.2025.1610093

**Published:** 2025-08-06

**Authors:** Zibai Guo, Jinhong Wei, Anping Gui, Xuanhua Liang, Pengli Yu, Jing Bai, Liang Cui, Xuefeng Xia, Shihui Ma

**Affiliations:** ^1^ Breast Center Zone One, Zhongshan City People’s Hospital, Zhongshan, China; ^2^ Medical Department, GenePlus-Beijing Institution, Beijing, China

**Keywords:** HER2-positive, breast cancer, neoadjuvant therapy, whole-genome methylation sequence, DNA methylation

## Abstract

**Background:**

Response to trastuzumab-based neoadjuvant therapy in human epidermal growth factor receptor type 2 (HER2)-positive breast cancer is affected by multiple features of the tumor. Few studies have investigated epigenetic features in these patients. This study investigates whether changes in deoxyribonucleic acid (DNA) methylation patterns are linked to response to neoadjuvant therapy in HER2-positive breast cancer and aims to identify epigenetic markers of treatment resistance.

**Methods:**

28 tumor samples were obtained from 20 HER2-positive breast cancer patients treated with neoadjuvant therapy: 12 from patients who achieved pathological complete response (pCR) before treatment, and 8 from patients who did not (non-pCR). For the non-pCR group, matched post-treatment samples were also collected, enabling paired pre- and post-treatment comparisons. After whole-genome methylation sequencing of all samples, the methylation differences between the pre-treatment pCR and non-pCR groups, as well as the methylation differences in non-pCR groups between pre-treatment and post-treatment samples were compared.

**Results:**

Before treatment, tumors in the non-pCR group showed slightly more hypomethylation events compared to the pCR group. After treatment, the same non-pCR tumors showed increased hypermethylation. Notably, immune-related pathways in these tumors were found to be hypermethylated, suggesting possible immune dysregulation. Methylation changes in the oncogenes *MOS* and *RET* were associated with potential resistance mechanisms. Additionally, four genes—*KIT*, *LAD1*, *FAM110C*, and *DAPP1*—were identified as candidate resistance markers based on their altered methylation patterns.

**Conclusions:**

These findings highlight how DNA methylation changes may influence treatment outcomes in HER2-positive breast cancer and suggest novel epigenetic markers that could help predict or overcome therapy resistance.

## Introduction

Human epidermal growth factor receptor type 2 (HER2)-positive breast cancer, which represents about 20–25% of all breast cancers, is more aggressive and prone to recurrence than other subtypes (1). Although HER2-targeted therapies (such as trastuzumab) have significantly improved survival, most patients eventually develop resistance—either from the beginning (primary) or over time (acquired)—which limits treatment success (2, 3).

Deoxyribonucleic acid (DNA) methylation is a chemical modification where a small molecule called a methyl group is added to DNA, often at specific sites (called CpG sites). This modification can turn genes on or off without changing the DNA sequence itself, and it plays an important role in health and disease (4, 5). In cancers, including breast cancer, abnormal methylation patterns can silence important tumor-suppressor genes. For instance, methylation of the *BRCA1* or *RASSF1A* gene promoters can inactivate these genes, contributing to tumor development (6, 7). Such methylation patterns may also influence tumor response to therapy.

Neoadjuvant therapy, which is treatment given before surgery, has improved outcomes for many HER2-positive breast cancer patients. However, drug resistance—whether present from the start (primary) or developing during treatment (acquired)—remains a significant obstacle. Understanding what causes resistance at the molecular level is critical for improving treatment responses (8, 9). However, the development of drug resistance limits the long-term efficacy of drugs. Tumor drug resistance is divided into primary drug resistance and acquired drug resistance. Primary drug resistance means that tumor cells are insensitive to drugs before treatment, while acquired drug resistance means that tumor cells gradually develop resistance to drugs during treatment (10). Some resistance may be driven by cancer stem cells (CSCs)—cells within tumors that can self-renew and survive treatment. These cells are regulated by pathways like STAT3 and Notch, which can also be affected by epigenetic changes, such as DNA methylation (11). *STAT3* signaling molecules and the Notch signaling pathway can promote the expression of stem cell markers and epithelial-mesenchymal transition (EMT), and play an important role in the Herceptin resistance process (12–14).

Although whole-genome DNA methylation characters of HER2-positive breast tumor tissues has been previously studied (4). Prediction of pathological complete response (pCR) among HER2-positive breast cancer patients receiving trastuzumab-based neoadjuvant therapy has also been conducted by integrated analysis of mRNA and DNA mutation in pretreatment tumors (15). Little is known so far about genome-wide methylation changes in these patients. The aim of this study was to determine DNA methylation changes that may influence treatment outcomes and identify probable epigenetic predictors related to neoadjuvant therapy.

## Methods

### Sample collection

Between May 2020 and June 2023, 20 patients diagnosed with malignant breast cancer who had sufficient tumor samples for whole-genome methylation sequence (GM-seq) were retrospectively enrolled in this study. The protocol was approved by the Ethics Committee of Zhongshan City People’s Hospital (K2023-198). Informed consent was obtained from all patients prior to sample collection and data use. ER/PR positivity in the immunohistochemistry testing were indicated with a cut-off of equal or higher than 1% positively stained cells. HER2 status was defined by an immunohistochemistry score of 3+ or presence of HER2 amplification (HER2/CEP17 ratio ≥ 2.0) demonstrated by fluorescence *in situ* hybridization (FISH) analysis. All patients received trastuzumab plus pertuzumab-based neoadjuvant therapy and subsequent radical surgery. pCR was defined as no evidence of the invasive cancer with or without intraductal components, including the lymph nodes, based on the pathological evaluation of the surgical breast specimen. A total of 28 perioperative tumor samples were obtained from 20 HER2-positive breast cancer patients treated with neoadjuvant therapy, including 12 pre-treatment samples from patients who achieved pCR, 8 pre-treatment samples and 8 matched post-treatment samples from 8 patients who did not (non-pCR).

### DNA extraction, library construction and GM-seq

Tissue DNA was extracted from FFPE by using QIAamp DNA MiniKit (Qiagen, Hilden, Germany) according to the manufacturer’s instructions. A total of 1 µg DNA was fragmented into 200–250 bp segments, using a Covaris S2 instrument (Woburn, MA, USA). DNA concentration was measured by Qubit™ dsDNA HS Assay Kit (ThermoFisher, Waltham, MA, USA).

We utilized the Hieff NGS Ultima Pro DNA Library Prep Kit for Illumina (Yeason, catalog number 12201ES96) for end repair, A-tailing, and adapter ligation. After magnetic bead purification, 5-methylcytosine (5mC) and 5-hydroxymethylcytosine (5hmC) are oxidized to 5-acylcytosine (5fC) or 5-carboxycytosine (5caC) by TET enzyme, and then used Pyridine borane treats 5fC or 5caC, reducing it to dihydrouracil (DHU) (16). Finally, PCR amplification was performed and barcodes were introduced to obtain a sequencing library. DNA libraries were assessed using Agilent Bioanalyzer. After this series of processes, the methylated cytosine was identified as thymine (T) by DNBSEQ-T7 (GenePlus-SuZhou, SuZhou, China) sequencing platform. Sequencing was performed with 150 bp paired-end reads, targeting >30 × coverage per sample. All libraries passed QC with >90% base quality score above Q30.

### Analysis of differentially methylated regions

The analysis of DMRs between groups was conducted using Metilene (v0.2–8) (17), and only data with a CpG coverage depth greater than 5 were included. DMRs were required to contain a minimum of 10 CpG sites with ≤300 bp spacing between adjacent sites. Significant DMRs were identified based on two criteria: (a) absolute methylation difference > 0.1 between groups, and (b) q-value < 0.05. Functional annotation of DMR-associated genes was performed through GO enrichment analysis using the online website Metascape (https://metascape.org/gp/index.html). Methylation patterns were visualized via hierarchical clustering with pheatmap (v1.0.12), employing Euclidean distance metrics.

### Gene promoter methylation

To ensure data reliability, only CpG sites with sequencing coverage ≥5× were retained for downstream genomic annotation. Gene promoter regions were defined as 1,000 bp upstream of transcription start sites (TSSs). Using BEDTools (v2.30.0), we extracted all qualifying CpG sites within these promoter regions. The promoter methylation level for each gene was then calculated as the mean β-value (methylation ratio = methylated reads/total reads) of all CpG sites within its promoter region. In the study, CpG sites were divided into three categories based on methylation levels: hyper (high methylation, beta value greater than 0.7), hypo (low methylation, beta value less than 0.3), and inter (moderate methylation, the rest of the range) (18).

### Statistical analysis

All analyses were performed using R v4.2.0. Statistical comparisons were two-sided and adjusted using false discovery rate (FDR) where appropriate. Data visualization was conducted using ggplot2 and Complex Heatmap packages. Comparisons between two groups were based on two-sided Wilcoxon rank-sum (Mann–Whitney U) test, and comparisons among multiple groups were based on Kruskal-Wallis test.

## Results

### Patient characteristics

20 female patients with stage II–III HER2-positive breast cancer were retrospectively enrolled in this study (**Table 1**). The median age of the study cohort was 48 years (range 32–68 years). Most patients were HR-positive and HER2-positive (65%) and all patients were clinically lymph node positive. All of the involved patients received trastuzumab plus pertuzumab-based neoadjuvant therapy and radical surgery. 12 (12/20, 60%)patients achieved pCR, while 8 (8/20, 40%)patients were non-pCR after neoadjuvant therapy.

**Table 1:** Characteristics of the patients.

Variables		*N* (%)
Age (years)	Median (range)	48 (32, 68)
Clinical tumor size (cT)	2	13 (65%)
	3	3(15%)
	4	4(20%)
Clinical nodal status (cN)	1	16(80%)
	2	3(15%)
	3	1(5%)
Stage	II	11(55%)
	III	9(45%)
Molecular subtypes	HR+HER2+	13(65%)
	HR-HER2+	7(35%)
pCR status	pCR	12(60%)
	Non-pCR	8(40%)

HR, hormone receptor; HER2, human epidermal growth factor; pCR, pathological complete response; non-pCR, non-pathological complete response.

### DNA methylation analysis in all tumors

**Figure 1:**
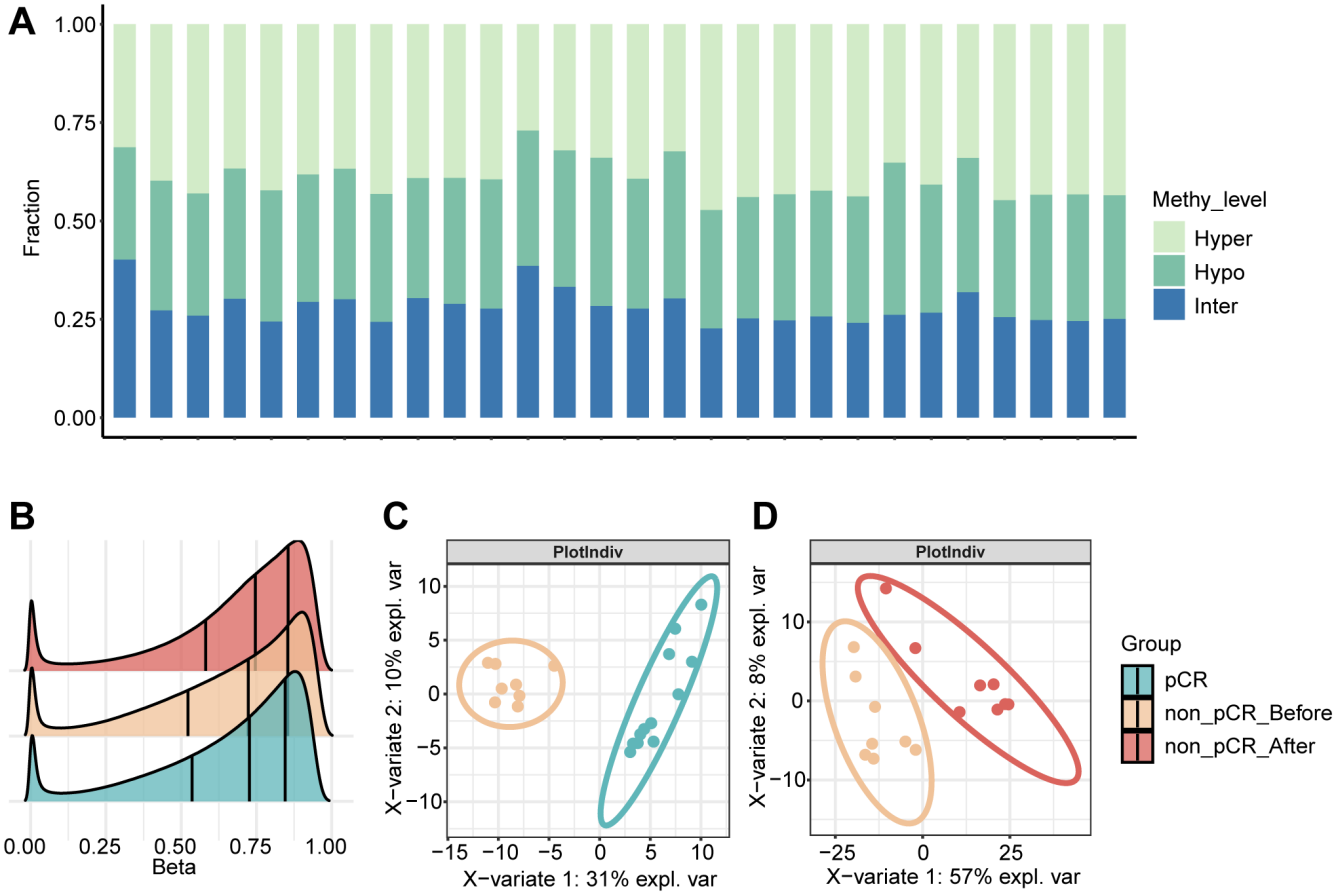
DNA methylation in HER2 positive breast tumors. **(A)** The proportional distribution of CpG methylation states (hyper/hypo/inter) across individual samples. **(B)** Density distribution of CpG methylation levels in pCR, non-pCR before and non-pCR after. **(C)** Partial least squares discriminant analysis (PLS-DA) based on the expression profiles of pCR vs. non-pCR before. **(D)** Partial least squares discriminant analysis (PLS-DA) based on the expression profiles of non-pCR before vs. non-pCR after. pCR, pathological complete response; non-pCR, non-pathological complete response.

We collected 28 *HER2*-positive breast cancer samples from 20 patients who underwent neoadjuvant therapy, including 12 pre-treatment samples from patients who achieved pCR, 8 pre-treatment samples and 8 matched post-treatment samples from 8 patients who did not (non-pCR). In order to further characterize the molecular characteristics of *HER2*-positive breast cancer treatment response, whole-genome methylation sequencing was performed on all the above samples using sufficient tissues. For the CpG sites in each sample, they were classified based on their methylation levels, with sites with beta values greater than 0.7 defined as hyper, sites with beta values less than 0.3 defined as hypo, and the rest defined as inter. The average proportion of hyper sites in all samples was 0.39, the average proportion of hypo sites was 0.35, and the average proportion of inter sites was 0.26 (**Figure 1A**). In the pre-treatment samples, the non-pCR group had slightly more hypomethylation events than the pCR group. In the paired non-pCR samples, the post-treatment group had more hypermethylation events than the pre-treatment group (**Figure 1B**). At the same time, the differentially methylated sites could not only clearly distinguish pCR and non-pCR samples, but also distinguish non-pCR patients before and after treatment (**Figures 1C, D**), emphasizing the potential impact of methylation changes on the treatment response of *HER2*-positive breast cancer.

### DNA methylation analysis in pCR and non-pCR groups before treatment

**Figure 2:**
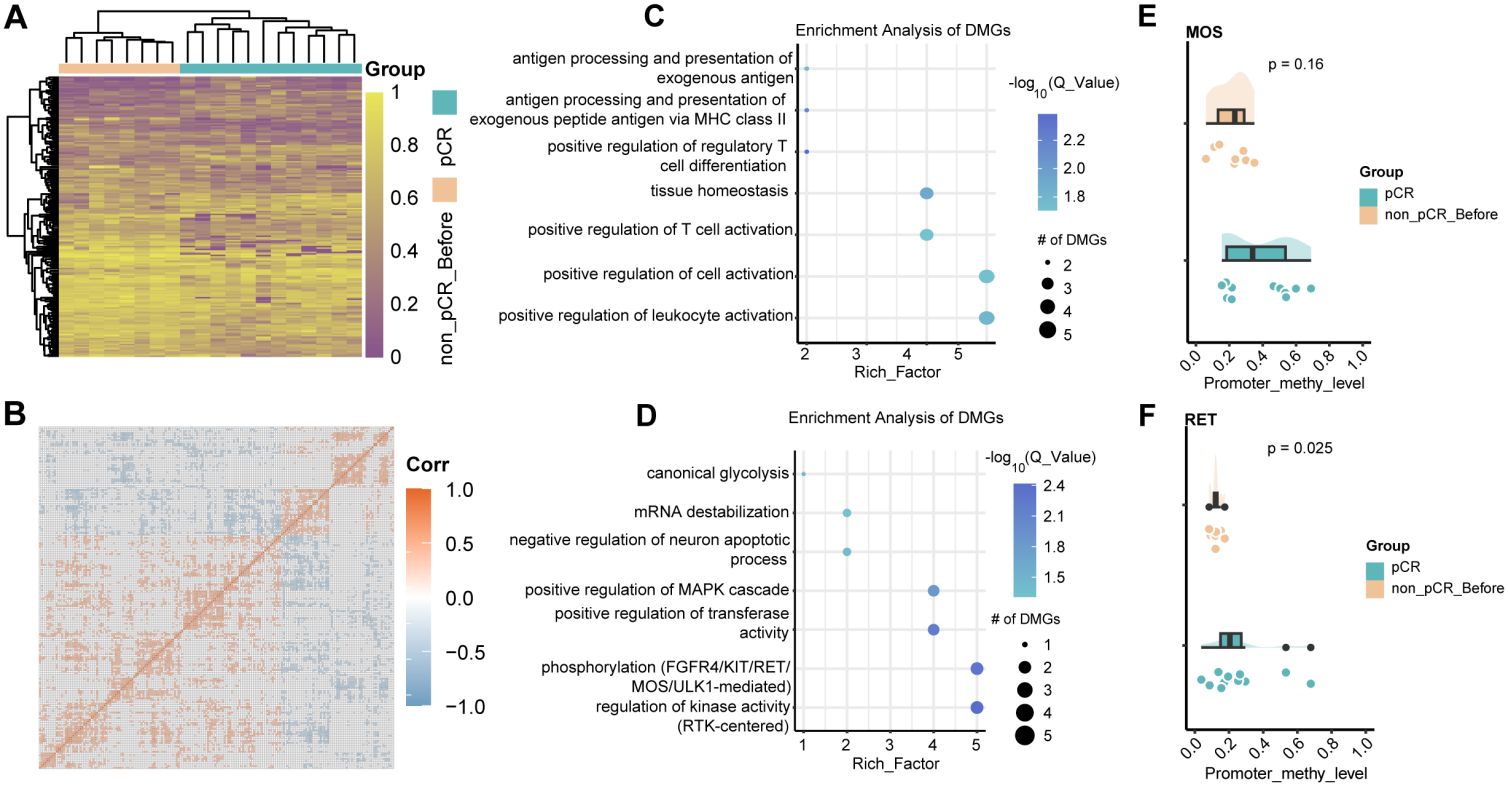
DNA methylation changes in HER2-positive breast cancer before treatment. **(A)** Heatmap visualization of methylation levels in differentially methylated regions between pCR and non-pCR before. Color gradient: purple (low methylation), yellow (high methylation). Significant DMRs: absolute methylation difference > 0.1 and q-value < 0.05. **(B)** Correlation of methylation in differentially methylated regions between pCR and non-pCR before. **(C)** Bubble plot visualization of GO BP pathway enrichment of hyper methylated genes in non-pCR before. **(D)** Bubble plot visualization of GO BP pathway enrichment of hypo methylated genes in non-pCR before. **(E)** Cloud-rain plot visualizes methylation levels of MOS promoter regions in pCR and non-pCR before. **(F)** Cloud-rain plot visualizes methylation levels of RET promoter regions in pCR and non-pCR before. pCR, pathological complete response; non-pCR, non-pathological complete response.

To further explore the methylation changes in the neoadjuvant therapeutic effect of HER2-positive breast cancer, we first performed differential methylation analysis in tumors before treatment. By comparing the tumors in the pCR and non-pCR groups, a total of 192 differentially methylated genes were identified. In non- pCR tumors, 61 hypomethylated and 131 hypermethylated gene promoters were identified separately. Hierarchical clustering analysis reveals distinct methylation profiles between the two groups, with non-pCR tumors exhibiting widespread hypermethylation compared to pCR tumors (**Figure 2A**). Correlation of methylation in differentially methylated regions showed that methylated genes in the same group tend to have co-hypermethylation or demethylation events (**Figure 2B**). The highly methylated genes in non-pCR tumors were enriched in multiple immune-related pathways, such as positive regulation of leukocyte activation, positive regulation of T cell activation, etc. (**Figure 2C**), which suggest that non-pCR tumors have immune disorders. Studies have shown that the HER2-L755S mutation can enhance *HER2* autophosphorylation and affect downstream MAPK and PI3K/AKT/mTOR signaling pathways (19). *ANKRD44* gene silencing activates *NF-κB* through the *TAK1*/*AKT* signaling pathway, and glycolysis levels increase in resistant cells (20). Interestingly, hypomethylated genes in non-pCR tumors were enriched in pathways such as phosphorylation (such as *FGFR4*, *KIT*, *MOS*, *RET* and *ULK1*) and canonical glycolysis (**Figure 2D**). This suggests that changes in gene promoter methylation may play a key role in the drug resistance mechanism that occurs with changes in different signaling pathways. Next, we focused our attention on oncogenes, which are genes that may be associated with the drug resistance mechanism. *MOS*, as a proto-oncogene, is also a serine/threonine kinase that activates the *MAP*K cascade by directly phosphorylating the *MAP* kinase activator *MEK*. Compared with pCR tumors, the level of methylation of the *MOS* gene in non-pCR tumors had a non-significant increasing trend (**Figure 2E**), suggesting that methylation variations in oncogenes may associated with the effect of treatment response. Another proto-oncogene that has attracted much attention is *RET*. Changes in the *RET* gene mainly include gene fusions and point mutations. Targeted therapies for *RET* gene changes mainly focus on *RET* kinase inhibitors, which mainly target *RET* gene fusions and certain point mutations, rather than methylation. However, changes in methylation cannot be ignored. In non-pCR tumors, there was a significantly lower methylation level, with a concentrated distribution and an average methylation level of only 0.17. In contrast, in pCR tumors, *RET* methylation levels were higher and relatively dispersed (**Figure 2F**). These phenomena may suggest that the correlation between treatment response and tumor methylation level.

### DNA methylation changes before and after treatment

**Figure 3:**
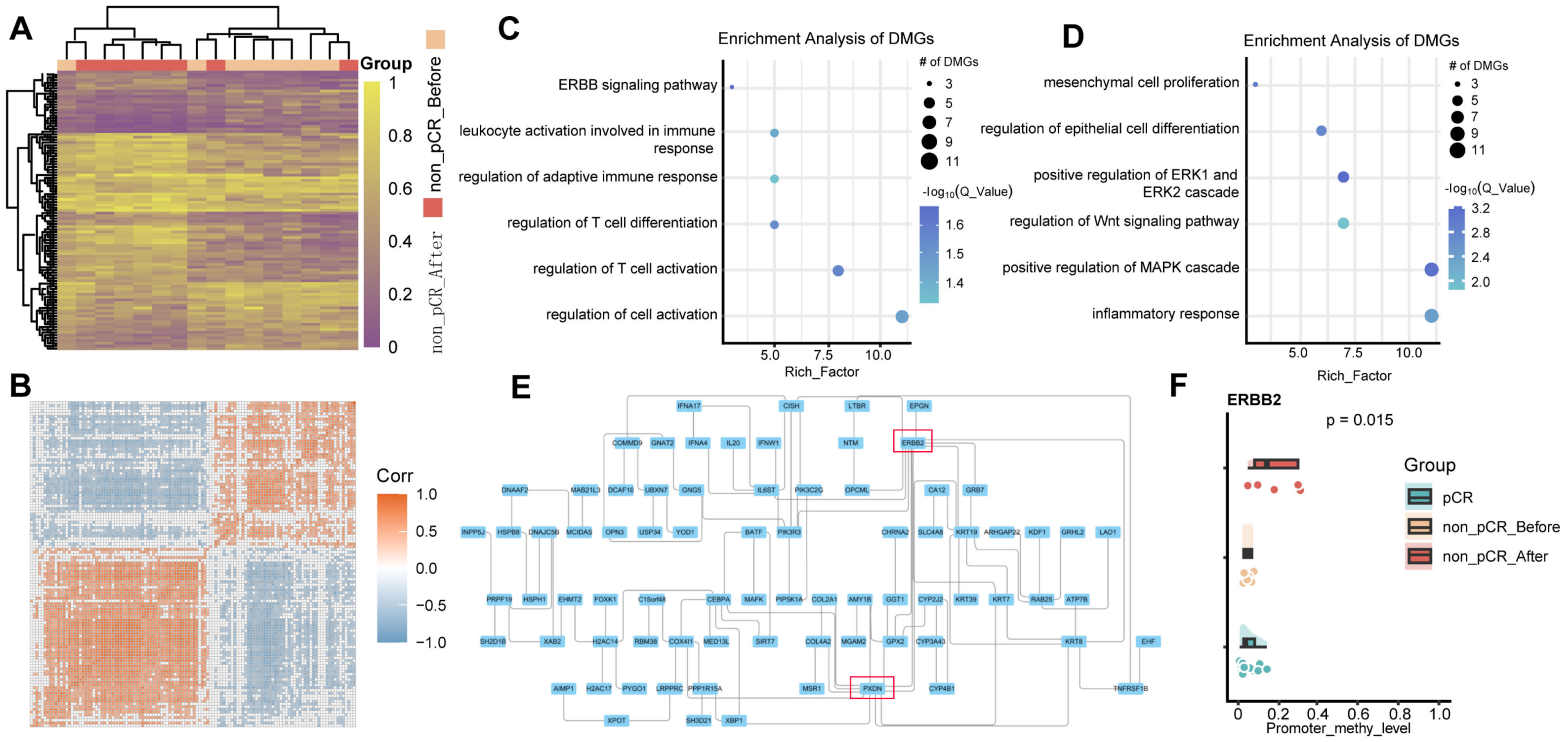
DNA methylation changes in non-pCR HER2-positive breast cancer before and after treatment. **(A)** Heatmap visualization of methylation levels in differentially methylated regions between non-pCR before and non-pCR after. Color gradient: purple (low methylation), yellow (high methylation). Significant DMRs: absolute methylation difference > 0.1 and q-value < 0.05. **(B)** Correlation of methylation in differentially methylated regions between non-pCR before and non-pCR after. **(C)** Bubble plot visualization of GO BP pathway enrichment of hyper methylated genes in non-pCR after treatment group. **(D)** Bubble plot visualization of GO BP pathway enrichment of hypo methylated genes in non-pCR after treatment group. **(E)** Interaction network of differentially hypermethylated genes in non-pCR before and non-pCR after. **(F)** Cloud-rain plot visualizes methylation levels of ERBB2 promoter regions in pCR, non-pCR before and non-pCR after. Non-pCR, non-pathological complete response.

Methylation changes within tumors after treatment still require further study. Therefore, we performed differential methylation analysis in paired samples before and after treatment in non-pCR patients. Compared with pre-treatment tumors, 284 significantly hypermethylated genes and 228 significantly hypomethylated genes were identified in tumors after treatment (**Figure 3A**). Similarly, methylated genes in the same group tend to undergo co-hypermethylation or demethylation events (**Figure 3B**). Among them, hypermethylated genes were involved in T cell activation, leukocyte activation, and other pathways involved in immune response (**Figure 3C**), while hypomethylated genes were involved in mesenchymal cell proliferation, positive regulation of *MAPK* cascade, and epithelial cell proliferation. (**Figure 3D**). These results demonstrated that treatment-resistant tumors might employ a biphasic epigenetic strategy: establishing immune privilege through hypermethylation of immunoregulatory genes while acquiring malignant potential via hypomethylation-activated mesenchymal and proliferative programs. To further understand the interactions between differentially methylated genes, we drew undirected interaction networks for hypermethylated and hypomethylated genes separately. It was worth noting that in the hypermethylated gene interaction network, we identified two key hub nodes, namely *ERBB2* and *PXDN* (**Figure 3E**). The *ERBB2* gene is a member of the epidermal growth factor receptor (*HER*) family and is related to a variety of cell signaling pathways that play important roles in cell proliferation, tumor formation, and apoptosis. In our study, no difference in *ERBB2* methylation was found between pCR and non-pCR pre-treatment tumors, but increased promoter methylation of *ERBB2* was found in non-pCR patients after treatment (**Figure 3F**). While increased promoter methylation may indicate gene silencing, this study does not directly measure ERBB2 expression, and further investigation is required. *PXDN* encodes a heme-containing peroxidase secreted into the extracellular matrix. It is involved in the formation of the extracellular matrix and may play a role in physiological and pathological fibrotic responses in the fibrotic kidney and can promote laminin assembly. Hypermethylation of Hub node genes may lead to decreased expression, thereby affecting downstream pathways. Hypermethylation of genes related to downstream pathways occurs at the same time, which further aggravates the drug resistance of tumors.

### Identification of potential resistance markers

**Figure 4:**
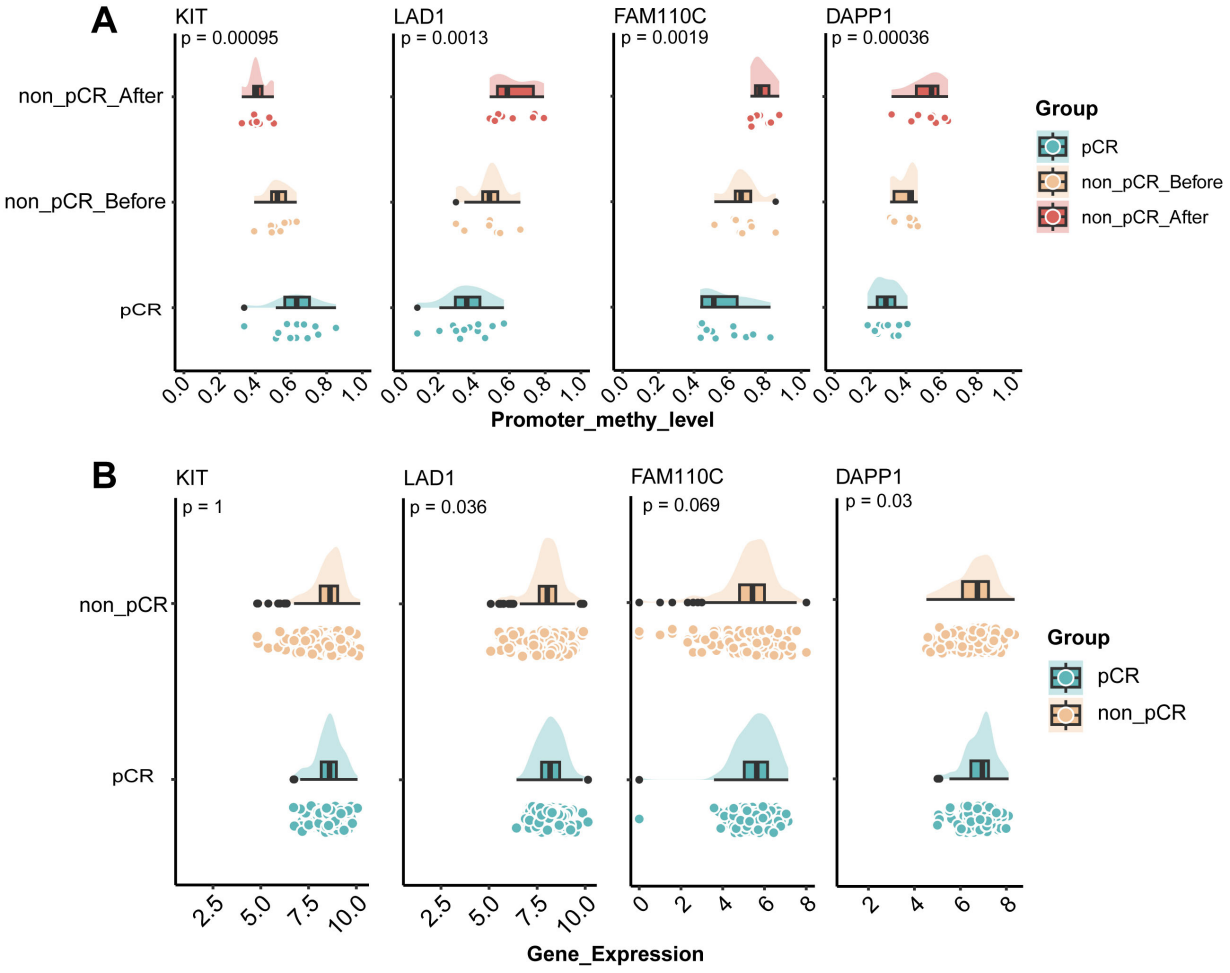
Identification of potential resistance markers in HER2-positive breast cancer. **(A)** Cloud-rain plot visualizes methylation levels of KIT, LAD1, FAM110C and DAPP1 promoter regions in pCR, non-pCR before and non-pCR after. **(B)** Cloud-rain plot visualizes gene expression of KIT, LAD1, FAM110C and DAPP1 in GSE243375 dataset between pCR and non-pCR group. pCR, pathological complete response; non-pCR, non-pathological complete response.

We hypothesized that genes exhibiting consistently increasing or decreasing promoter methylation levels across the three comparison groups (pCR, non-pCR before, and non-pCR after) would facilitate the identification of reliable resistance biomarkers. Therefore, we integrated the differentially methylated genes between pCR before treatment and non-pCR after treatment, and collected the RNA-sequencing profile of 285 *HER2*-positive breast cancer tumors receiving neoadjuvant therapy in GSE243375 dataset for verification. Finally, four genes with the same methylation difference trend were identified, namely *KIT*, *LAD1*, *FAM110C*, and *DAPP1*. As shown in **Figure 4A**, *KIT* exhibited progressively decreasing methylation levels in treatment-resistant cases, representing the only gene with this declining trend. In contrast, *LAD1* (p=0.0013), *FAM110C* (p=0.0019) and *DAPP1* (p=0.00036) all showed significant gradual methylation increases among the three groups. Notably, *LAD1*-encoded basement membrane anchoring protein showed reduced expression in poor responders (**Figure 4B**, p=0.036). Similarly, *DAPP1* - related to innate immunity - exhibited both progressively increased methylation and downregulated expression (**Figure 4B**, p=0.03). These findings suggested that the methylation levels of genes related to epithelial-mesenchymal homeostasis and immune environment homeostasis may affect the treatment response of *HER2*-positive breast cancer.

## Discussion

In this study, we used whole-genome methylation sequencing to characterize the methylation patterns of 28 *HER2*-positive breast cancer tissues, including 12 samples before neoadjuvant treatment in pCR group, 8 samples before treatment and paired 8 samples after surgery in non-pCR group. Our study showed that non-pCR tumors had a widespread hypermethylation pattern. Hypermethylation affected gene promoter regions, especially members of oncogenes, immune pathways, and epithelial-mesenchymal transition pathways. In addition, we found 4 genes whose methylation differences were further amplified after treatment.

When comparing pre-treatment tumors with different efficacy, it was found that genes with low methylation in non-pCR tumors were enriched in phosphorylation-related pathways (*FGFR4, KIT, MOS, RET, ULK1*). And when comparing the methylation of non-pCR tumors before and after treatment, it was found that the enrichment of low-methylation genes in the post-treatment group was also enriched in the positive regulation pathway of phosphorylation. *Rack1* promotes the phosphorylation of *Anxa2* by *Src* by mediating the interaction between Src and Anxa2, thereby promoting the increase in the migration and invasion ability of drug-resistant cells (21). *NNMT* (nicotinamide methyltransferase) is highly expressed in drug-resistant tumors and low in sensitive tumors; *DNMT1* (DNA methyltransferase 1) is low in drug-resistant tumors and high in sensitive tumors (22). *NNMT* and *DNMT1* jointly maintain the sensitivity of tumor cells to oxidative phosphorylation inhibitors (23). In addition, it was found that the promoter region of the *FOXK1* which is related to glycolysis was hypomethylated. Hypomethylation may correspond to the existence of high expression, which makes glycolysis more active. Glycolysis not only provides energy for tumor cells, but also provides the raw materials required for biosynthesis, promoting the rapid proliferation of tumors (24). Studies have shown that the enhancement of glycolytic activity is related to tumor progression and drug resistance (25). For example, key enzymes and metabolites in the glycolysis process can affect tumor progression and drug resistance through various mechanisms, including inhibiting cell apoptosis, promoting epithelial-mesenchymal transition (EMT) of tumor cells, and inducing autophagy (26). The discovery of *FOXK1* may provide a new therapeutic strategy for overcoming tumor resistance by intervening in the glycolysis pathway.

The highly methylated genes in non-pCR tumors are enriched in immune-related pathways. When comparing the methylation of non-pCR tumors before and after treatment, it was found that the enrichment of highly methylated genes in the post-treatment group was also enriched in immune-related pathways. This phenomenon may indicate that tumor cells develop multiple mechanisms to promote their immune escape. Identifying tumor-intrinsic factors that support immune escape may provide new strategies for cancer immunotherapy. Internal factors of the tumor itself will prevent immune cell infiltration or suppress the immune microenvironment, making it difficult for immunotherapy to work, leading to the occurrence of immune resistance (27).

Among the differentially methylated genes identified, changes in proto-oncogenes also attracted our attention. Genes such as *MOS* and *RET* are hypomethylated in non-pCR tumors. The *RET* signaling network has been shown to affect multiple axes of breast cancer, including tumor development, metastasis, and treatment resistance (28). *RET* overexpression is associated with tumorigenesis and endocrine therapy resistance (29). Our study provides a new probable direction in epigenetic modification for inhibiting resistance. Due to limitations in sample size, these findings require validation in larger cohorts.

Existing methylation differences within the tumor may affect the treatment effect, and these differences may be further amplified as the treatment progresses, thereby continuously affecting the treatment effect (15). In this study, we found 4 genes (*KIT, LAD1, FAM110C, DAPP1*) whose methylation differences were further amplified after treatment. The *KIT* encodes a receptor tyrosine protein kinase and is a proto-oncogene that acts as a cell surface receptor for the cytokine *KITLG*/*SCF* and plays a vital role in regulating cell survival and proliferation, hematopoiesis, stem cell maintenance, gametogenesis, mast cell development, migration and function, and melanogenesis (30). The *KIT* gene is the only gene with a continuous decrease in methylation. Although there are many variants of *KIT* mutants and they do not show completely consistent biological characteristics, most of them have high *KIT* expression levels (31). It is worth noting that the high expression level of the *KIT* gene is not related to its gene amplification (32). Promoter methylation may be a key factor in regulating *KIT* expression (33). *DAPP1* is one of the genes with a continuously increased methylation level. However, studies have shown that the lack of *DAPP1* can promote anti-tumor response and increase the expression of Tim3 in *CD8*+ T cells (34). This may indicate that methylation is not the keyway to regulate *DAPP1* to affect drug resistance, and it is still necessary to expand the sample size and conduct complex and comprehensive research on genomic and transcriptomic data.

This study has some limitations. First, due to retrospective nature of this study, some patients had insufficient tumor tissue for GM-seq, thus patients included in the analysis were limited. Additionally, due to the nature of our study design and the availability of samples, we were only able to analyze tumor samples. Patients in pCR groups were unable to provide tumor tissue for analysis. Second, biomarkers discovered in the study need further *in vitro* and *in vivo* validation.

Our study provides the first evidence that genome-wide methylation patterns in HER2-positive breast cancer may help predict treatment outcomes. Methylation signatures like those seen in *KIT* and *DAPP1* may serve as biomarkers or therapeutic targets to overcome resistance. Further research is needed to translate these findings into clinical practice.

## Data Availability

The original contributions presented in the study are publicly available. This data can be found here: https://ngdc.cncb.ac.cn/gsa-human, accession number HRA012294.

## References

[1] Jerez Y Herrero B Arregui M Moron B Martin M Echavarria I. Neratinib for the treatment of early-stage, hormone receptor-positive, HER2-overexpressed breast cancer. Future Oncol. (2020) 16:1165–77. doi: 10.2217/fon-2020-0046, PMID: 32458702

[2] Loibl S Gianni L. HER2-positive breast cancer. Lancet. (2017) 389:2415–29. doi: 10.1016/S0140-6736(16)32417-5, PMID: 27939064

[3] Oh DY Bang YJ. HER2-targeted therapies - a role beyond breast cancer. Nat Rev Clin Oncol. (2020) 17:33–48. doi: 10.1038/s41571-019-0268-3, PMID: 31548601

[4] Lindqvist BM Wingren S Motlagh PB Nilsson TK. Whole genome DNA methylation signature of HER2-positive breast cancer. Epigenetics. (2014) 9:1149–62. doi: 10.4161/epi.29632, PMID: 25089541 PMC4164500

[5] Huang YT Li FF Ke C Li Z Li ZT Zou XF. PTPRO promoter methylation is predictive of poorer outcome for HER2-positive breast cancer: indication for personalized therapy. J Transl Med. (2013) 11:245. doi: 10.1186/1479-5876-11-245, PMID: 24090193 PMC3852714

[6] Yu J Zayas J Qin B Wang L. Targeting DNA methylation for treating triple-negative breast cancer. Pharmacogenomics. (2019) 20:1151–7. doi: 10.2217/pgs-2019-0078, PMID: 31755366 PMC7026764

[7] Strelnikov VV Kuznetsova EB Tanas AS Rudenko VV Kalinkin AI Poddubskaya EV. Abnormal promoter DNA hypermethylation of the integrin, nidogen, and dystroglycan genes in breast cancer. Sci Rep. (2021) 11:2264. doi: 10.1038/s41598-021-81851-y, PMID: 33500458 PMC7838398

[8] Piccart-Gebhart MJ Procter M Leyland-Jones B Goldhirsch A Untch M Smith I. Trastuzumab after adjuvant chemotherapy in HER2-positive breast cancer. N Engl J Med. (2005) 353:1659–72. doi: 10.1056/NEJMoa052306, PMID: 16236737

[9] Romond EH Perez EA Bryant J Suman VJ Geyer CE Jr. Davidson NE. Trastuzumab plus adjuvant chemotherapy for operable HER2-positive breast cancer. N Engl J Med. (2005) 353:1673–84. doi: 10.1056/NEJMoa052122, PMID: 16236738

[10] Tan Y Sun R Liu L Yang D Xiang Q Li L. Tumor suppressor DRD2 facilitates M1 macrophages and restricts NF-kappaB signaling to trigger pyroptosis in breast cancer. Theranostics. (2021) 11:5214–31. doi: 10.7150/thno.58322, PMID: 33859743 PMC8039962

[11] Tolaney SM Tayob N Dang C Yardley DA Isakoff SJ Valero V. Adjuvant trastuzumab emtansine versus paclitaxel in combination with trastuzumab for stage I HER2-positive breast cancer (ATEMPT): A randomized clinical trial. J Clin Oncol. (2021) 39:2375–85. doi: 10.1200/JCO.20.03398, PMID: 34077270

[12] Andre F Ismaila N Allison KH Barlow WE Collyar DE Damodaran S. Biomarkers for adjuvant endocrine and chemotherapy in early-stage breast cancer: ASCO guideline update. J Clin Oncol. (2022) 40:1816–37. doi: 10.1200/JCO.22.00069, PMID: 35439025

[13] Tomasich E Steindl A Paiato C Hatziioannou T Kleinberger M Berchtold L. Frequent overexpression of HER3 in brain metastases from breast and lung cancer. Clin Cancer Res. (2023) 29:3225–36. doi: 10.1158/1078-0432.CCR-23-0020, PMID: 37036472

[14] Hu Y Liu S Cui C Liu X Li H Liu H. Enhanced HER2 status detection in breast and gastric cancers using surrogate DNA methylation markers. IUBMB Life. (2025) 77:e70004. doi: 10.1002/iub.70004, PMID: 39988770

[15] Tanioka M Fan C Parker JS Hoadley KA Hu Z Li Y. Integrated analysis of RNA and DNA from the phase III trial CALGB 40601 identifies predictors of response to trastuzumab-based neoadjuvant chemotherapy in HER2-positive breast cancer. Clin Cancer Res. (2018) 24:5292–304. doi: 10.1158/1078-0432.CCR-17-3431, PMID: 30037817 PMC6214737

[16] Chen X Liu J Li J Xie Y Yu Z Shen L. Identification of DNA methylation and genetic alteration simultaneously from a single blood biopsy. Genes Genomics. (2023) 45:627–35. doi: 10.1007/s13258-022-01340-y, PMID: 36512197

[17] Jühling F Kretzmer H Bernhart SH Otto C Stadler PF Hoffmann S. metilene: fast and sensitive calling of differentially methylated regions from bisulfite sequencing data. Genome Res. (2016) 26:256–62. doi: 10.1101/gr.196394.115, PMID: 26631489 PMC4728377

[18] Holm K Staaf J Lauss M Aine M Lindgren D Bendahl PO. An integrated genomics analysis of epigenetic subtypes in human breast tumors links DNA methylation patterns to chromatin states in normal mammary cells. Breast Cancer Res. (2016) 18:27. doi: 10.1186/s13058-016-0685-5, PMID: 26923702 PMC4770527

[19] Li J Xiao Q Bao Y Wang W Goh J Wang P. HER2-L755S mutation induces hyperactive MAPK and PI3K-mTOR signaling, leading to resistance to HER2 tyrosine kinase inhibitor treatment. Cell Cycle. (2019) 18:1513–22. doi: 10.1080/15384101.2019.1624113, PMID: 31135266 PMC6592242

[20] La Ferla M Lessi F Aretini P Pellegrini D Franceschi S Tantillo E. ANKRD44 gene silencing: A putative role in trastuzumab resistance in Her2-like breast cancer. Front Oncol. (2019) 9:547. doi: 10.3389/fonc.2019.00547, PMID: 31297336 PMC6607964

[21] Simonova OA Kuznetsova EB Tanas AS Rudenko VV Poddubskaya EV Kekeeva TV. Abnormal hypermethylation of CpG dinucleotides in promoter regions of matrix metalloproteinases genes in breast cancer and its relation to epigenomic subtypes and HER2 overexpression. Biomedicines. (2020) 8. doi: 10.3390/biomedicines8050116, PMID: 32397602 PMC7277193

[22] Ding Y Liu Y Lee DK Tong Z Yu X Li Y. Cell lineage tracing links ERalpha loss in Erbb2-positive breast cancers to the arising of a highly aggressive breast cancer subtype. Proc Natl Acad Sci U.S.A. (2021) 118. doi: 10.1073/pnas.2100673118, PMID: 34006643 PMC8166171

[23] Nikolaienko O Eikesdal HP Ognedal E Gilje B Lundgren S Blix ES. Prenatal BRCA1 epimutations contribute significantly to triple-negative breast cancer development. Genome Med. (2023) 15:104. doi: 10.1186/s13073-023-01262-8, PMID: 38053165 PMC10698991

[24] Liu Q Kulak MV Borcherding N Maina PK Zhang W Weigel RJ. A novel HER2 gene body enhancer contributes to HER2 expression. Oncogene. (2018) 37:687–94. doi: 10.1038/onc.2017.382, PMID: 29035388 PMC5794618

[25] Palomeras S Diaz-Lagares A Vinas G Setien F Ferreira HJ Oliveras G. Epigenetic silencing of TGFBI confers resistance to trastuzumab in human breast cancer. Breast Cancer Res. (2019) 21:79. doi: 10.1186/s13058-019-1160-x, PMID: 31277676 PMC6612099

[26] Collins DM Madden SF Gaynor N AlSultan D Le Gal M Eustace AJ. Effects of HER family-targeting tyrosine kinase inhibitors on antibody-dependent cell-mediated cytotoxicity in HER2-expressing breast cancer. Clin Cancer Res. (2021) 27:807–18. doi: 10.1158/1078-0432.CCR-20-2007, PMID: 33122343 PMC7854527

[27] Tolaney SM Goel S Nadal J Denys H Borrego MR Litchfield LM. Overall Survival and Exploratory Biomarker Analyses of Abemaciclib plus Trastuzumab with or without Fulvestrant versus Trastuzumab plus Chemotherapy in HR+, HER2+ Metastatic Breast Cancer Patients. Clin Cancer Res. (2024) 30:39–49. doi: 10.1158/1078-0432.CCR-23-1209, PMID: 37906649 PMC10767303

[28] Jacobs SA Wang Y Abraham J Feng H Montero AJ Lipchik C. NSABP FB-10: a phase Ib/II trial evaluating ado-trastuzumab emtansine (T-DM1) with neratinib in women with metastatic HER2-positive breast cancer. Breast Cancer Res. (2024) 26:69. doi: 10.1186/s13058-024-01823-8, PMID: 38650031 PMC11036567

[29] Liu R Liu L Zhao C Bai Y Zheng Y Zhang S. Larotinib in patients with advanced and previously treated esophageal squamous cell carcinoma with epidermal growth factor receptor overexpression or amplification: an open-label, multicenter phase 1b study. BMC Gastroenterol. (2021) 21:398. doi: 10.1186/s12876-021-01982-4, PMID: 34688250 PMC8540164

[30] Sheikh E Tran T Vranic S Levy A Bonfil RD. Role and significance of c-KIT receptor tyrosine kinase in cancer: A review. Bosn J Basic Med Sci. (2022) 22:683–98. doi: 10.17305/bjbms.2021.7399, PMID: 35490363 PMC9519160

[31] Paroni G Bolis M Zanetti A Ubezio P Helin K Staller P. HER2-positive breast-cancer cell lines are sensitive to KDM5 inhibition: definition of a gene-expression model for the selection of sensitive cases. Oncogene. (2019) 38:2675–89. doi: 10.1038/s41388-018-0620-6, PMID: 30538297

[32] von der Heyde S Wagner S Czerny A Nietert M Ludewig F Salinas-Riester G. mRNA profiling reveals determinants of trastuzumab efficiency in HER2-positive breast cancer. PloS One. (2015) 10:e0117818. doi: 10.1371/journal.pone.0117818, PMID: 25710561 PMC4339844

[33] Gao E Wang X Wang F Deng S Xia W Wang R. Systematic characterization of expression patterns and immunocorrelations of formin-like genes in breast cancer. BioMed Res Int. (2022) 2022:8577821. doi: 10.1155/2022/8577821, PMID: 36124068 PMC9482526

[34] Mudvari P Ohshiro K Nair V Horvath A Kumar R. Genomic insights into triple-negative and HER2-positive breast cancers using isogenic model systems. PloS One. (2013) 8:e74993. doi: 10.1371/journal.pone.0074993, PMID: 24086418 PMC3781103

